# How accurate is probabilistic tractography when used to predict the “sweet spot” in deep brain stimulation? Mind the gap!

**DOI:** 10.1007/s00701-025-06719-w

**Published:** 2025-12-01

**Authors:** Daniel Deuter, Tobias Mederer, Katharina Rosengarth, Judith Anthofer, Anes Dada, Michael Knott, Tina Wendl, Nils-Ole Schmidt, Jürgen Schlaier

**Affiliations:** 1 Klinik und Poliklinik für Neurochirurgie, University Hospital Regensburg, Franz-Josef-Strauß-Allee 11, 93053 Regensburg, Germany; 2 Institut für Röntgendiagnostik, University Hospital Regensburg, Franz-Josef-Strauß-Allee 11, 93053 Regensburg, Germany; 3 Institut für Neuroradiologie, Medbo Bezirksklinikum Regensburg, Universitätsstraße 84, 93053 Regensburg, Germany

**Keywords:** Probabilistic tractography, Deep Brain Stimulation, Diffusion Tensor Imaging, Fiber tracts

## Abstract

**Background:**

Tractography has been used in various studies with respect to the improvement of patient-specific DBS targeting. Nevertheless, methodological influences of the chosen parameters and associated errors are often neglected. The aim of this study was to estimate concrete errors associated with specific image processing steps when using measurements of distances to specific subcortical fiber tracts to predict optimal stimulation sites for DBS targeting.

**Method:**

Probabilistic tractography of the crossing and non-decussating part of the dentato-rubro-thalamic-tract (c-/ nd-DRTT) was performed using FSL 6.0.3 in 40 PD- and ET-patients having received bilateral DBS surgery. DBS-electrodes were reconstructed using LeadDBS. The influence of (1) the choice of threshold for binarization of fiber tracts, (2) manual measurements compared to measurements using automized distance maps and (3) normalization into the MNI standard space on measured distances were investigated.

**Results:**

Different thresholds for binarization resulted in non-linear and unpredictable variations of measured distances up to 1.72 ± 1.49 mm (mean value ± standard deviation). Manual measurements on the axial slice of the electrode contact showed a mean error of 0.91 ± 1.36 mm (maximum 14.9 mm) compared to automated measurements. Regarding normalization, a mean error of 0.82 ± 0.50 mm (maximum 2.34 mm) was found compared to measurements in native space.

**Conclusion:**

Measured maximum errors reach up to several millimeters, which might have significant impact on clinical targeting in DBS. Researchers should be aware of these errors and define individual standards for specific studies.

## Introduction

Tractography has been used in a variety of studies to predict the “sweet spot” for the DBS electrode in various diseases [16, 23, 74]. Current pathophysiological models assume a mediation of DBS effects via fibers travelling through the volume of tissue electrically activated (VTA) evoking effects also distant to the electrode pole [2, 7, 10, 12, 28, 40, 67]. Therefore, tractography is more and more discussed also with respect to tractographically defined patient-specific targets [16, 18, 23].

Tractography is based on Diffusion Tensor Imaging (DTI), depicting the diffusion of water molecules, which is usually aligned to the course of white matter fiber tracts [79]. While deterministic tractography is based on only one main diffusion vector for each imaging voxel, probabilistic tractography rather rests on probability distributions of multiple diffusion vectors for each voxel [15, 63, 73, 79]. Using probabilistic tractography, it is therefore possible to delineate crossing, fanning and kissing fibers as well as intersecting and bending fibers [25, 63, 73].

Within the scope of DBS, in literature, mainly three approaches have been applied:

1. Analysis of distances of the electrode poles to a specific fiber tract [1, 19, 24, 26, 27],
2. Visualization of fibers associated with specific electrode poles using these poles as seedregions for tractography [58, 70, 71] culminating in whole-brain connectomic approaches [2, 12, 40, 76] and
3. Connectivity-based localization of specific targets not yet identifiable based on conventional magnetic resonance imaging (MRI) like the ventral intermediate nucleus of the thalamus (VIM) [4, 60].

Increasingly, normalization of patient cohorts into a standard space like the Montreal Neurological Institute (MNI) space is performed because these aggregated data enable easier comparisons and visualizations of larger groups on one single standard brain [30, 41, 43, 55]. Especially when discussing probabilistic tractography with respect to patient-specific targeting, a robust evaluation of associated errors seems inevitable. But as workflows for tractography consist of a multitude of steps and a vast heterogeneity exists between workflows, final and conclusive error assessment remains difficult. Nevertheless, an estimation of specific errors associated with single image processing steps is highly relevant, especially regarding the clinical applicability of tractographic findings for the neurosurgical definition of patient-specific DBS targets.

**Fig. 1:**
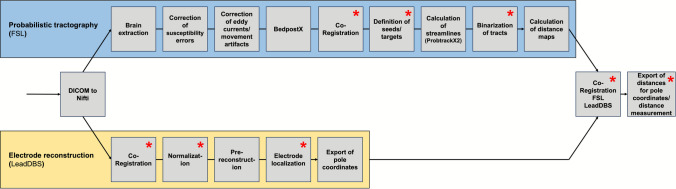
Overview of the computational workflow: The flowchart shows the specific steps of the workflow for probabilistic tractography using FSL (blue box) and for electrode reconstruction using LeadDBS (yellow box). The red * marks steps of the workflow identified potentially bearing relevant source of error as these are not fully automated, specific parameters have to be defined or manual manipulations or checks of accuracy are necessary. For specific FSL protocols like TOPUP, standardized parameters have successfully been described, which most of the time don’t have to be manually adapted. Therefore, these steps were not considered for error assessment in the following

The aim of this study was to assess the concrete errors associated with specific steps of the workflow when analyzing the position of the DBS electrode in relation to a specific fiber tract. Figure 1 shows the computational workflow used for probabilistic tractography and calculation of distances to the DBS electrodes including specific processing steps identified as potentially bearing relevant source of error. In this study, we focus on the impact of the choice of threshold used for the binarization of fiber tracts, the technique used for measurements of distances (manual measurements vs. measurements using automated distance maps) and normalization into the MNI standard space on measured distances.

## Methods and materials

This study was approved by the local ethics committee of the University Hospital of Regensburg, Germany (protocol code Z-2017–0876-10) and was performed in accordance with the Declaration of Helsinki. We analyzed the influence of specific steps of the workflow on the distances of the electrode poles to the crossing and non-decussating part of the dentato-rubro-thalamic tract (c-DRTT/nd-DRTT), which has previously been discussed with respect to tremor reduction in ET and PD [1, 19, 20, 24, 26, 27, 32, 33, 52, 64]. For probabilistic tractography and further calculations, FSL [68, 77] and LeadDBS [42] were used as commonly applied in literature. We retrospectively analyzed a cohort of 40 patients (24 male, 16 female) having undergone DBS surgery at the Center for Deep Brain Stimulation of the University of Regensburg between 2015 and 2020. The cohort consisted of 22 PD patients having bilaterally received DBS surgery with implantation of electrodes into the Subthalamic Nucleus (STN) and 18 ET patients with implantation of electrodes into the VIM.

In the following, we first describe the workflow regarding imaging and clinical setup, probabilistic tractography and reconstruction of DBS electrodes. Second, the methodological workaround used for the assessment of errors associated with the examined steps of the workflow is presented. In this analysis, we investigated (1) the influence of the choice of threshold for the binarization of fiber tracts on measured distances, (2) errors associated with manual distance measurements compared to automated measurements and (3) normalization into the MNI standard space.

### Workflow for probabilistic tractography and reconstruction of DBS-electrodes

#### Imaging and clinical setup

Preoperatively, MR imaging was performed at a 3 Tesla MRI scanner (Siemens Skyra, Siemens Healthineers, Erlangen, Germany). In order to avoid movement artifacts, images were acquired with patients under general anesthesia. For planning of the stereotactic target, imaging included sagittal T1 sequences, axial and sagittal T2 images aligned parallel to the intercommissural plane and T1 + one and a half times dose of Gadolinium to depict crucial blood vessels. Imaging parameters were previously described by Strotzer et al. [71]. DTI sequences were acquired with 64 gradient directions. The voxel size of DTI sequences was 2.0 × 2.0 × 2.0 mm. Only patients for whom antiparallel b-zero images were acquired for the estimation of susceptibility-induced errors were included into this study. A stereotactic Computed Tomography (CT) was acquired using a Siemens Somatom Definition Flash scanner (Siemens Healthineers) on the day of surgery with a CRW frame (Integra Radionics, Burlington, VT) attached to the patient’s head for planning of the stereotactic trajectory. Targeting was performed as previously described in [27] for ET patients and in [26] for PD patients. Postoperatively, CT scans with 1 mm slice thickness were performed to verify correct electrode positions.

#### Probabilistic tractography (FSL)

Probabilistic tractography of the c-DRTT and the nd-DRTT was performed using FSL 6.0.3 [68, 77] as summarized in Table 1. For the definition of the threshold to binarize the fiber tracts based on their probability maps, we calculated thresholds defined as 400%, 600%, 800%, and 1000% of the “robust range” (“-thrp”) of the histogram, as these parameters showed the best results in previous tests. The “robust range” focusses on the values between the 2% and 98% percentile of the histogram ignoring extreme values [37]. Subsequently, binarized tracts, based on these thresholds, were opened in parallel and overlaid on the T2-weighted images. The threshold leading to the best discrimination between the c-DRTT and nd-DRTT was chosen as the final threshold. As this final threshold was chosen for the patient’s whole brain and not for a specific hemisphere, sometimes compromises between the two hemispheres had to be taken. Finally, distance maps from the binarized tracts were calculated using ANTs 2.2.0 [8] evoking image maps, where each voxel represents the shortest distance to the specific tract as a value.

**Table 1:** Workflow for probabilistic tractography using FSL 6.0.3

	Processing step	Specific algorithm implemented in FSL
1	Brain extraction	BET [69]
2	Correction of susceptibility-induced errors	TOPUP [5, 68]
3	Correction of eddy current-induced aberrations and proband movements	EDDY[6]
4	Calculation of voxel-specific probability distributions of diffusion orientations based on models of multiple diffusion orientations for each imaging voxel	BedpostX [36], based on an algorithm described by Behrens et al. [15]
5	Co-registration of T2-weighted images and DTI sequences	FSL FLIRT [48, 49]
6	Definition of seed and target regions on T2-weighted images	FSLeyes [53]: Seed = red nucleus (RN) including the lateral interspace towards the STN, target c-DRTT = contralateral superior cerebellar peduncle (SCP), target nd-DRTT = ipsilateral SCP
7	Iterative calculation of streamlines interconnecting the specific seed and target regions culminating in track probability maps	ProbtrackX2 [36]: Number of samples = 5000, step length = 0.5 mm, curvature threshold = 0.2, maximum number of steps = 2000, subsidiary fiber volume threshold = 0.01, minimum length threshold = 0, seed sphere sampling = 0
8	Binarization of fiber tracts	

The table shows the specific processing steps and the computational algorithms used. *c-DRTT* = crossing part of the dentato-rubro-thalamic tract, *DTI* = Diffusion Tensor Imaging, *nd-DRTT* = non-decussating part of the dentato-rubro-thalamic tract, *RN* = Red nucleus, *SCP* = Superior cerebellar peduncle, *STN* = Subthalamic nucleus

#### Electrode reconstruction (LeadDBS)

Electrodes were reconstructed using LeadDBS [42]. Table 2 summarizes the specific steps of the workflow and the algorithms used. For each electrode, coordinates of the electrode poles were exported using Matlab 2018a (MathWorks, Natick, MA) from the LeadDBS dataset in the native space. These coordinates were visually controlled to be located on the electrode in the postoperative CT scan to check for the correctness of the export.

**Table 2:** Workflow for the reconstruction of DBS electrodes using LeadDBS

	Processing step	Specific algorithm implemented in LeadDBS
1	Co-registration	CT to MRI: ANTs [9], MRI to MRI: SPM [35] or FSL FLIRT [48, 49]
2	Normalization into the MNI standard space	ANTs three-step affine normalization [9, 34, 65]
3	Electrode pre-reconstruction	PaCER [44]
4	Manual localization of electrodes	
5	Visualization of results in native patient space	

The table shows the specific processing steps and the computational algorithms used. *CT* = Computed Tomography, *MNI* = Montreal Neurological Institute, *MRI* = Magnetic resonance imaging

To enable comparisons between the FSL datasets and the data from LeadDBS, the anatomic T2 scan (FSL) was co-registered to the postoperative CT scan (LeadDBS) using FSL FLIRT [48, 49]. For this co-registration, a transformation matrix was exported and applied to all results from probabilistic tractography transferring these into the LeadDBS space. For each electrode pole coordinate, we exported the distances to the c-DRTT and the nd-DRTT of the specific hemisphere using the individually calculated distance maps. If distances exceeded 10 mm, thresholds for the individual tract were switched one step lower to achieve a more realistic representation of the specific tract.

### Methodological impact and estimation of error regarding specific steps of the workflow

#### Choice of threshold for binarization of fiber tracts

By now, no commonly accepted approach exists for the decision on how to define the threshold used for binarization of specific fiber tracts. To check how the distances to specific tracts are influenced, distances to the c-DRTT and nd-DRTT were exported for all the above calculated thresholds 400, 600, 800 and 1000% of the “robust range” within our chosen method of standardized thresholding (definition of only one threshold for the c-DRTT and nd-DRTT based on optimal discriminability between the two tracts on a whole-brain base, but not for each hemisphere). The possible impact of the usage of other methods for standardized thresholding as previously discussed in [27], for example based on the definition of a fixed value for each tract, was not part of this study.

#### Influence of manual distance measurements

To evaluate possible errors due to manual distance measurements performed on axial slices, we calculated the distance of each electrode pole to its closest point on the c-DRTT and nd-DRTT manually and based on automated distance maps. For manual measurements, axial slices were exported for the coordinate of each electrode pole. Euclidean distances between the pole coordinate and the coordinates of the closest point on the c-DRTT and the nd-DRTT were calculated for each pole on the axial slice. Additionally, distances obtained by the measurements using the automated distance maps as described above, were exported. Differences between automatically and manually measured distances were calculated.

#### Normalization into the MNI standard space

To assess errors associated with normalization into the MNI standard space, individual patients’ data were normalized as described above using the ANTs three-step affine normalization algorithm. To enable a comparison of distance measurements in MNI and the native space, we measured the distance of the tip of the electrode to the red nucleus (RN) in both spaces, as this structure can be easily and reliably identified in both the individual patient’s imaging and the MNI space. MR-coordinates were acquired in the native space at the anterior border of the RN on the axial slice with its biggest anterior–posterior expansion as well as at the tip of the DBS electrode. Additionally, corresponding coordinates were acquired for the RN using the DISTAL atlas [30] and the tip of the DBS electrode in MNI space. We calculated euclidean distances between the coordinates for both spaces and determined the individual differences between the measured distances in the MNI and the native space.

## Results

### Choice of threshold for binarization of fiber tracts

**Fig. 2:**
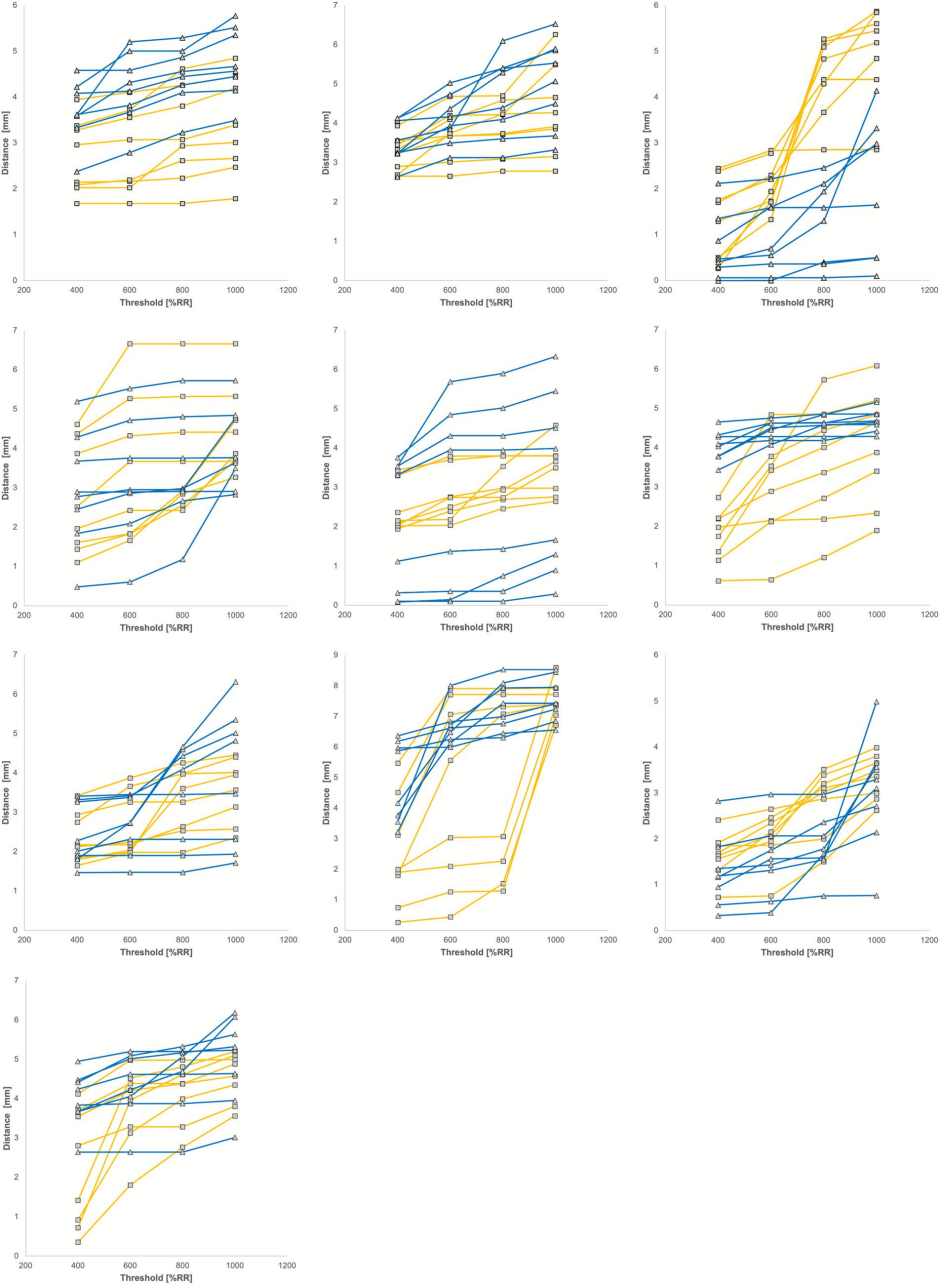
Correlation of the measured distances and chosen thresholds in a subgroup of 10 randomly chosen patients (ET N = 5, PD N = 5): Distances to the c-DRTT (blue, Δ) and the nd-DRTT (yellow, ∎) determined using automated distance maps are shown for each electrode contact in mm (y-axis) plotted against the chosen threshold in % of the “robust range” (x-axis)

**Fig. 3:**
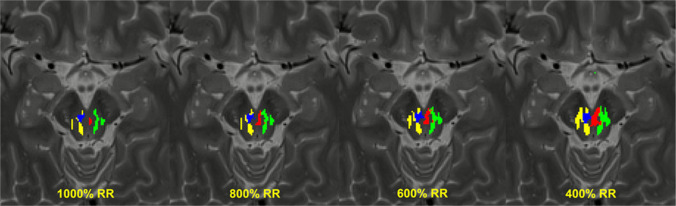
Expansion of the fiber tracts of interest (c-DRTT and nd-DRTT) depending on the chosen threshold. Binarized fiber tracts are shown for an exemplary patient on an axial T2 image above the decussation of the c-DRTT and below the Red Nucleus using 1000%, 800%, 600% and 400% of the “robust range” as thresholds for binarization. Red: Left c-DRTT, blue: right c-DRTT, green: left nd-DRTT, yellow: right nd-DRTT

In the analyzed cohort, a relevant influence of the different thresholds used for binarization was found. As different thresholds for binarization led to non-linear influences difficult to predict, we only present the results from a subgroup of 10 randomly chosen patients (ET N = 5, PD N = 5) in the following as shown in Fig. 2 for visualization convenience. Regarding a possible correlation between the chosen threshold and the distances to the specific electrode poles, no predictable pattern could be found for either the different electrode poles within patients as well as between patients. Figure 3 shows the expansion of the fiber tracts at interest depending on the chosen threshold shown on the anatomic T2 images in an exemplary patient. Maximum differences between the measurements based on all analyzed thresholds in this subgroup were on average 1.72 ± 1.49 mm (mean value ± standard deviation) with a maximum error of 6.60 mm. It should be kept in mind that this metric error does not really reflect the accuracy of thresholding as it is mainly dependent on the span of investigated thresholds. A larger span would result in larger errors (if examining for example thresholds between 400% and 10,000% of the “robust range” rather than between 400 and 1000%), a smaller span in smaller errors. Nevertheless, this number gives a slight hint on the magnitude how big the influence of thresholding can be, even if in reality, some of these thresholds might not be considered rational due to anatomic reasons when visually inspecting the results in relation to the T2 images.

### Influence of the technique of distance measurements

Manual measurement was not possible for 78 distances as the specific tract was not evident on the axial slice through the electrode contact. This leads to a total of 578 measured distances (40 patients with 328 electrode contacts and distances to 2 tracts each). The mean difference between manual axial distance measurements compared to measurements using automatically calculated 3D distance maps was 0.91 ± 1.36 mm (mean value ± standard deviation) with a maximum aberration of 14.9 mm.

### Normalization into the MNI standard space

In two cases misrepresentation of coordinates after normalization was assumed due to relevant discrepancy between native and normalized images (N = 2, mean differences of measured distances in MNI and native space 6.02 and 22.91 mm). After exclusion of these cases, a mean difference of 0.82 ± 0.50 mm (mean value ± standard deviation) was found between measurements in MNI and native space with a maximum error of 2.34 mm.

A summary of the measured errors associated with each of the three investigated processing steps is shown in Table 3.

**Table 3:** Summary of measured errors

	Mean error ± standard deviation	Maximum error
Choice of threshold for binarization of fiber tracts	1.72 ± 1.49 mm	6.60 mm
Influence of the technique of distance measurements	0.91 ± 1.36 mm	14.9 mm
Normalization into the MNI standard space	0.82 ± 0.50 mm	2.34 mm

Mean errors ± standard deviation as well as maximum errors are shown for the three investigated processing steps. *MNI* = Montreal Neurological Institute

## Discussion

In this study, we analyzed the influence of specific steps included in the workflow for probabilistic tractography on measured distances of the DBS electrode poles to specific fiber tracts. As tractography is discussed more and more with regard to patient-specific DBS-targeting and also the definition of new DBS targets, concrete estimations of the levels of error are highly relevant regarding the clinical applicability of tractographic results, but also their reproducibility on an individual basis. We analyzed the influence of the choice of the threshold for binarization of fiber tracts and the influence of manual measurements compared to measurements using automated distance maps as well as normalization into the MNI standard space enabling normative measurements and analyses on aggregated data. Nevertheless, the aim of this study was not to provide a conclusive and final error assessment of probabilistic tractography in general. In the following, we discuss the three analyzed aspects thresholding, technique of distance measurements as well as normalization each. Afterwards, limitations and additional sources of error, that were not part of the study, are presented.

### Thresholding

In our analysis, a profound influence of the threshold on the measured distances was found. Additionally, no specific patterns could be identified regarding the influence of the threshold on measured distances within patients as well as between patients. As these influences also compromise comparability of results between different study groups, the specific way of thresholding should be specifically reported. We previously discussed the issue of how to define the thresholds to binarize fiber tracts for further analyses. At least four decisions have to be made for a standardized definition of thresholds [27]:

- Definition of the general method of thresholding (for example conventional thresholding, thresholding based on a fixed percentage of the “robust range” [37], usage of an arbitrarily fixed threshold, usage of a fixed percentage rate of the minimum or maximum, …)
- Definition of the method how to numerically specify the threshold within the chosen general method (usage of a fixed percentage rate of the minimum or maximum, usage of a fixed threshold, …)
- Definition of the method how thresholds are related to each other if more than one tract is analyzed (usage of a fixed threshold for each tract independently of other tracts, usage of individual thresholds for each tract focused on proper differentiation between tracts like the c-DRTT and nd-DRTT, usage of a fixed value for all of the tracts, …)
- Definition of the method which reference is used if more than one tract is analyzed and if thresholds are chosen to be related to each other (definition of a threshold for the c-DRTT and the nd-DRTT with reference on a whole-brain base, usage of only the tracts of one hemisphere as reference, …).

In this study, the numerical choice of the threshold led to non-linear influences on the measured distances, difficult to predict. An analysis of the additional influence of the choice of the method how to define the thresholds was not part of this study. Previously, also more sophisticated approaches like distance-dependent thresholding were proposed [17]. Nevertheless, by now, no accepted standard exists for threshold definition. Therefore, specific methodological standards should be defined on an individual basis for each project which at least ensures comparability between results regarding specific tracts within one study.

### Technique of distance measurements

Various authors used manual measurements on axial planes or euclidean distances in preceding studies (see for example [1, 20, 72]). In our analysis, a direct comparison between manual measurements on the axial plane of the electrode pole contact and automated distance measurements led to a mean error of 0.91 mm, but with a maximum deviation of 14.9 mm between both methods. Though, no three-dimensional manual measurements were performed nor measurements at the nearest point on also coronal or sagittal slices, which would probably lower the expected error compared to measurements on only axial slices. Anyway, the importance of objective measurements, for example based on the usage of automatically calculated and investigator-independent distance maps, has to be clearly underlined, especially with regard to the complex spatial courses of specific fiber tracts.

### Normalization

A group of authors previously underlined the risk of using normative data as a basis for surgical decision making [21]. In our study, we found a mean difference of 0.82 mm between normalized data and measurements in the native patient space when measuring the distance of the tip of the DBS electrode to the RN with a maximum error of 2.34 mm after exclusion of cases where misrepresentation of coordinates was assumed. Ewert et al. previously compared automated atlas segmentations in the MNI standard space with manual segmentations as well as the inter-rater variability using two independent experts. For the automated methods, they found median Dice coefficients between 0.56 (linear) and 0.74 (ANTs SyN) compared to manual segmentations. These results were comparable to the inter-rater variability, which was found to be in median 0.78 in a cohort of healthy subjects, even if the concrete influence on specific distances remains unclear. The authors underline the variability of results regarding the different algorithms and nicely show how suboptimal normalizations might lead to false assumptions in relation to the position of the DBS electrode (see Ewert et al., Fig. 6) [31]. In another study, Vogel et al. [75] found Dice coefficients between 0.682 and 0.691 using ANTs for non-linear atlas creation performing intra-atlas assessments. As we wanted to assess concrete influences on the measured distances, but not measures of image similarity on datasets in general, we did not use Dice coefficients or other measures of image similarity in this study. A few other studies also focus on the influence of normalization with respect to normative connectivity profiles [76], DWI data [45] as well as tractographic results [3]. In most of the studies, no absolute errors regarding concrete distances to specific tracts were analyzed as performed in this study. The mean error of 0.82 mm seems to be generally acceptable regarding the analysis of aggregated datasets using huge cohorts for hypothesis-building studies. However, respecting the maximum error of 2.34 mm, results from aggregated datasets should be thoroughly checked and critically reviewed. As in the field of DBS targeting, distances of only several millimeters highly influence clinical effects, the analysis of data on an individual basis in the native patient space has still to be regarded as gold-standard and should be used especially in small cohorts and if final neurosurgical targets should be defined in individual patients.

### Limitations and additional sources of error that were not part of this study

Within this study, we focused only on three major sources of error, namely thresholding, the influence of manual distance measurements as well as normalization. As it was not the aim of this study to finally assess the specific error of tractography in general, we investigated the impact of several steps, which are often underestimated or even ignored with respect to possible errors. Because for specific FSL protocols like TOPUP, standardized parameters have successfully been described, which often don’t have to be adapted, the influence of these parameters was not part of the study. Also, imaging aspects like the impact of the number of gradient directions etc. were not part of the analysis as well as co-registration, the definition of seed and target regions and electrode localization. Furthermore, we did not assess the influence of specific algorithms, for example for normalization, and compared these methods to each other. However, these factors could significantly contribute to total error. Table 4 summarizes specific steps of the computational workflow bearing the risk of additional error on top of the investigated processing steps.

**Table 4:** Additional sources of error associated with specific steps of the workflow used in this study

Part of the workflow	Processing step	
Preprocessing	MR imaging parameters and scanner effects including the number of acquisition directions	[61]
Processing	Co-registration of CT- and MR-images	[13, 38, 54, 56]
Tractography/FSL	Parameters used for preprocessing steps like TOPUP and EDDY and for fiber tracking	
	Definition of seed and target regions for fiber tracking (ROIs)	
	Usage of algorithms for deterministic or probabilistic tractography	[57, 63, 80]
	Usage of different software packages	
Electrode reconstruction/LeadDBS	Algorithm for electrode reconstruction	[44, 78]
	Algorithms for the correction of postoperative brain shift	[14]
	Models how VTAs are calculated if used for the analyses	[7, 28]

The table shows the specific processing steps as well as their position within the workflow. *CT* = Computed Tomography; *MRI* = Magnetic resonance imaging; *ROI* = Region of Interest; *VTA* = Volume of Tissue electrically activated

With special respect to co-registration, several authors evaluated the influence of the image fusion between CT and MRI images [13, 38, 54, 56]. Regarding concrete metrical errors, Barnaure et al. found errors between 0.17 and 0.97 mm in a cohort of 23 DBS patients using a commercially available software package. Satisfactory results were reached using automatic alignment in only 70% of the cases [13]. Geevarghese et al. found a mean error of 0.72 mm in another 20 patients [38], a further study found errors between 1.2 and 1.7 mm depending on the used software algorithm [56]. As this specific step of image processing works on an automated basis, special attention should be paid at a comprehensive check of the results.

To conclude, it should be kept in mind that maximum errors sum up over the workflow, consisting of a multitude of steps, and cumulatively interfere. Several studies aiming for validation of specific tractographic approaches thus performed comparisons between different tractographic methods [11, 39, 61, 62] or compared tractographic results to a ground truth from clinical data like electrophysiology [22, 46, 59], animal models [29, 50] or histology [66]. Nevertheless, despite these efforts, a final and conclusive estimation of the level of error associated with specific tractographic approaches in general can’t be performed yet.

## Conclusions

Various authors previously discussed the possibilities and pit-falls of tractographic techniques, but only partially from a neurosurgical point-of-view [47, 51, 79]. We investigated the concrete influences of specific steps performed during standard workflows on the measured distances to specific fiber tracts. These parameters are often underestimated or even ignored when using probabilistic tractography with respect to DBS-related questions. As errors in the workflow sum up and interfere, including errors, which were not part of this study, special attention should be paid to these factors, especially in the field of DBS requiring precisions of only several millimeters. To ensure the applicability of tractographic results and also their reproducibility on an individual basis, specific standards should be defined by authors, at least enabling solid comparisons between fiber tracts within single studies, and transparently reported.

## Data Availability

Availability of raw datasets supporting the findings of this study is limited due to ethical reasons as these are containing information which could possibly compromise the privacy of research participants. Further inquiries can be directed to the corresponding author (DD).

## Abbreviations

CT: Computed Tomography
c-DRTT: Crossing part of the dentato-rubro-thalamic tract
DBS: Deep Brain Stimulation
DTI: Diffusion Tensor Imaging
ET: Essential Tremor
GPi: Internal part of the globus pallidus
MNI: Montreal Neurological Institute
MRI: Magnetic resonance imaging
nd-DRTT: Non-decussating part of the dentato-rubro-thalamic tract
PD: Parkinson’s Disease
RN: Red nucleus
ROI: Region of Interest
SCP: Superior cerebellar peduncle
STN: Subthalamic nucleus
VIM: Ventral intermediate nucleus of the thalamus
VTA: Volume of Tissue electrically activated

## Declarations

All procedures were performed in compliance with relevant laws and institutional guidelines. All experiments were performed in accordance with the Declaration of Helsinki. The study has been approved by the local ethics committee of the University Hospital of Regensburg, Germany (protocol code Z-2017–0876-10).
